# Cecal obstruction due to primary intestinal tuberculosis: a case series

**DOI:** 10.1186/1752-1947-5-128

**Published:** 2011-03-30

**Authors:** Antonis Michalopoulos, Vassilis N Papadopoulos, Stavros Panidis, Theodossis S Papavramidis, Anastasios Chiotis, George Basdanis

**Affiliations:** 1 First Propedeutic Department of Surgery, AHEPA University Hospital, Aristotle University of Thessaloniki, Thessaloniki, Greece

## Abstract

**Introduction:**

Primary intestinal tuberculosis is a rare variant of tuberculosis. The preferred treatment is usually pharmaceutical, but surgery may be required for complicated cases.

**Case presentation:**

We report two cases of primary intestinal tuberculosis where the initial diagnosis was wrong, with colonic cancer suggested in the first case and a Crohn's disease complication in the second. Both of our patients were Caucasians of Greek nationality. In the first case (a 60-year-old man), a right hemicolectomy was performed. In the second case (a 26-year-old man), excision was impossible due to the local conditions and peritoneal implantations. Histopathology revealed an inflammatory mass of tuberculous origin in the first case. In the second, cell culture and polymerase chain reaction tests revealed *Mycobacterium tuberculosis*. Both patients were given anti-tuberculosis therapy and their post-operative follow-up was uneventful.

**Conclusions:**

Gastrointestinal tuberculosis still appears sporadically and should be considered in the differential diagnosis along with other conditions of the bowel. The use of immunosuppressants and new pharmaceutical agents can change the prevalence of tuberculosis.

## Introduction

Based on surveillance and survey data, the World Health Organization (WHO) estimates that 9.27 million new cases of tuberculosis occurred in 2007. Primary intestinal tuberculosis (PITB) is a rare variant of the disease accounting for 1% of the cases in Europe [1]. Primary tuberculosis of the colon (PTBC) is nowadays rarely seen in Western countries and sporadic cases are present in the international bibliography. The rarity of PTBC is not only due to the rarity of *Mycobacterium tuberculosis *in general, but also because of the difficulty in identifying it in the biopsies taken by endoscopy. It is estimated that only one out of three cases of lower gastrointestinal tuberculosis gives a positive identification of the mycobacterium by culture, and two out of three cases by polymerase chain reaction (PCR) [2]. However it remains a considerable diagnostic challenge, especially in the absence of pulmonary infection, as it may mimic many other abdominal diseases such as infectious processes, tumors, peri-appendiceal abscesses and Crohn's disease (CD) [3-5]. The differential diagnosis between Crohn's disease (CD) and PTBC is crucial, because of the different treatment approaches, especially with regard to the use of immunomodulators and biological agents. One must also emphasize the need for clinical doctors to have a high awareness of the disease, especially in an era where demographic facts change constantly.

In this report, we present two cases with primary PTBC. The initial diagnosis suggested in the first case was colonic cancer, and in the second a complication of CD.

## Case presentation

### Case 1

**Figure 1:**
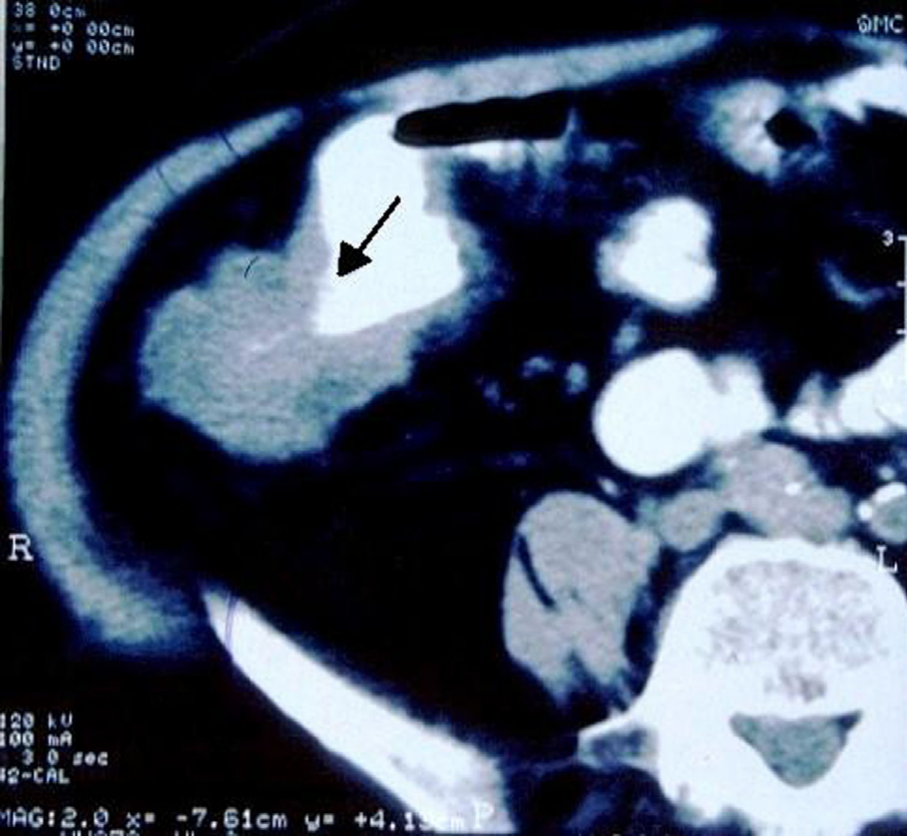
**Abdominal computed tomography revealing the site of the obstruction**.

**Figure 2:**
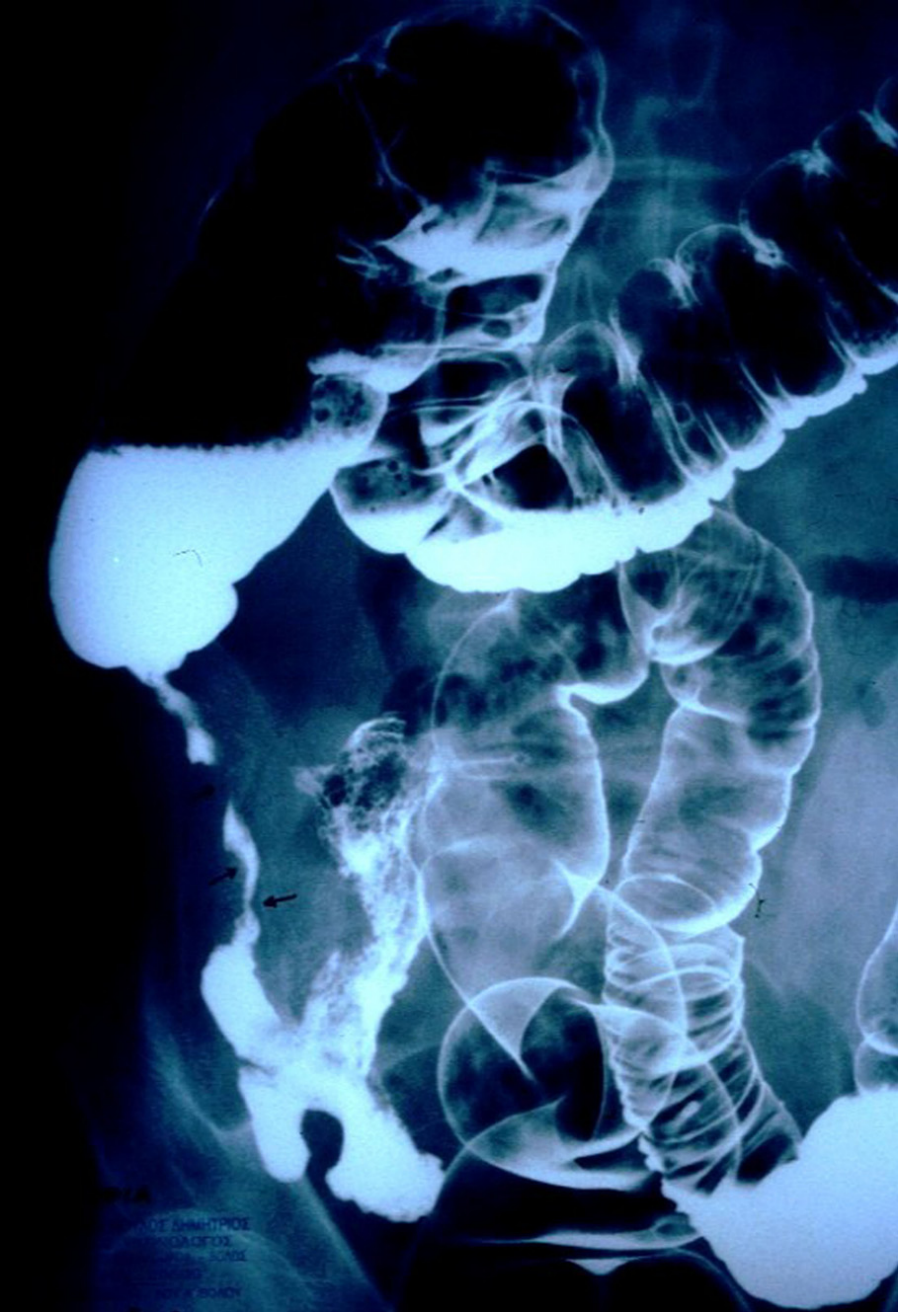
**Double contrast barium enema revealing a stricture in the region of the ileo-cecal valve and ascending colon**.

A 60-year-old Greek Caucasian man was referred to our emergency department with acute abdominal pain of the lower right quadrant. He mentioned gradual weight loss during the past few months. A physical examination revealed mild tenderness and a palpable mass in the right ileac fossa. Laboratory test findings showed mild anemia (hematocrit 33%, hemoglobin 10 mg/dL), a white blood cell count of 8000 cells/mm^3^, and mild hypoalbuminemia (3.0 g/dL). Liver and kidney functions were within normal range, and results of a chest X-ray were unremarkable. During his hospitalization, he presented with low fever (37.1°C to 37.6°C) and complained of deterioration of his abdominal pain. A contrast-enhanced computed tomography (CT) scan was performed, and revealed a mass located in the region of the cecal valve (Figure 1). A double-contrast barium enema was performed, revealing a stricture in the region of the ileo-cecal valve and ascending colon, which caused the obstructive phenomena (Figure 2). Colonoscopy was not available.

**Figure 3:**
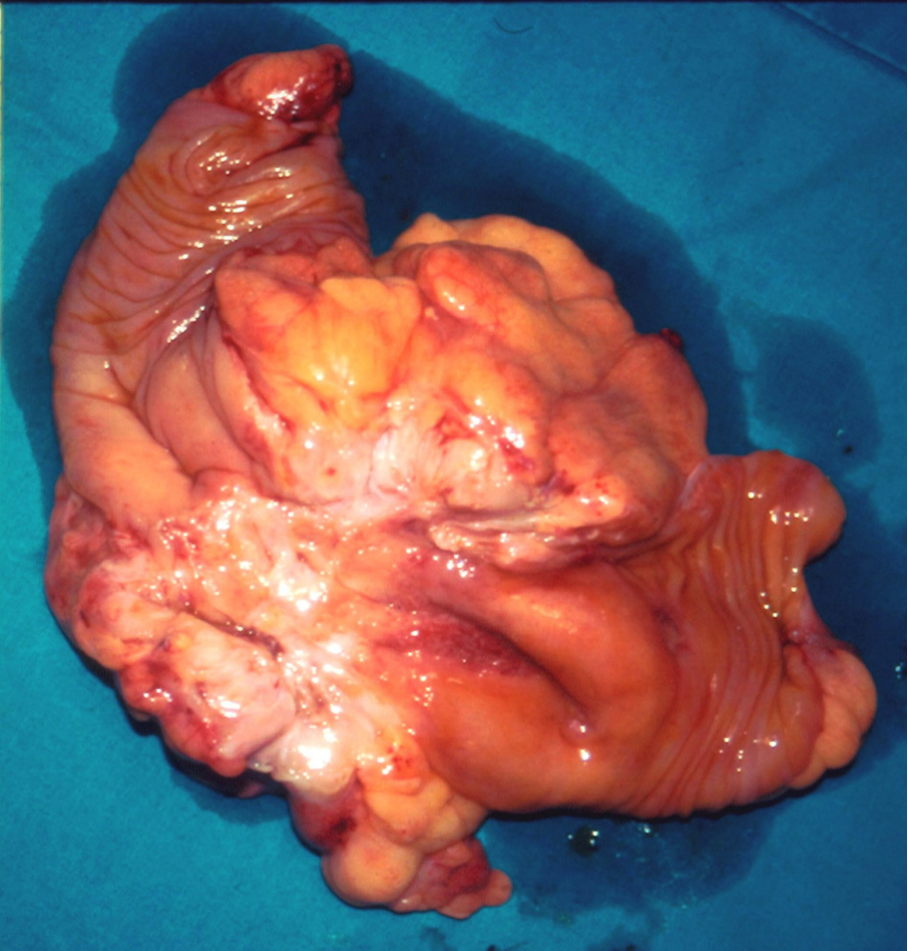
**Tubercular mass of the cecum**.

A typical right hemicolectomy was performed (Figure 3) and the pathological examination revealed intestinal tuberculosis. After this final diagnosis our patient received rifampicin 500 mg/day and isoniazid 330 mg/day for six months, and pyrizinamide 25 mg/kg daily for the first two months. Today, eight years after the operation, our patient remains disease free as proven by regular radiological follow-up.

### Case 2

**Figure 4:**
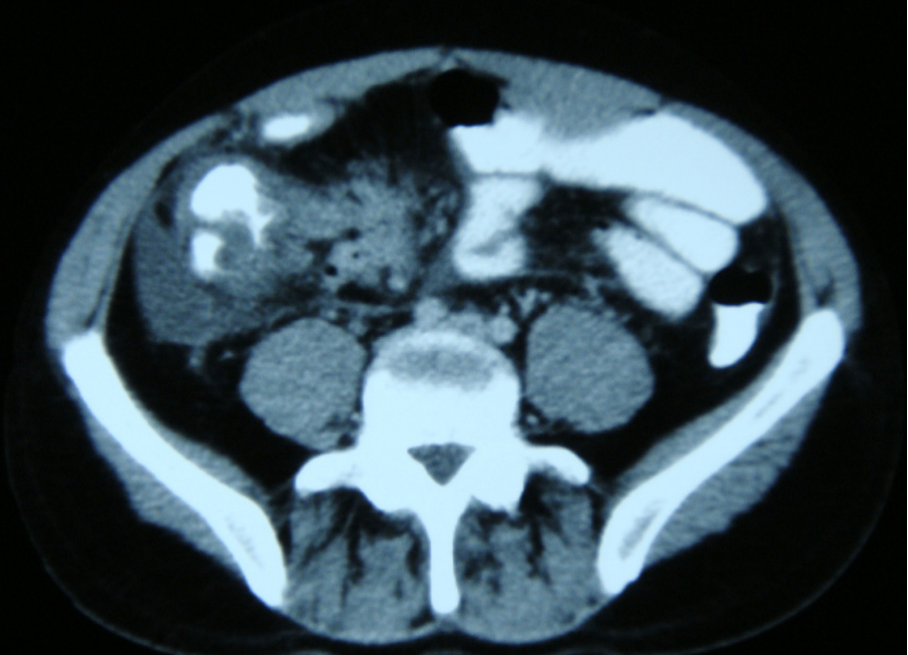
**Contrast-enhanced abdominal computed tomography showing the cecal mass**.

A 26-year-old Greek Caucasian man was referred to our out-patient department with episodes of abdominal pain, loss of weight, fever, anorexia and general weakness for the past six months. He had a history of CD from the age of 19, and he was being treated with infliximab (5 mg/kg). During the past six months he had been admitted twice to other hospitals with the same symptoms and discharged with the diagnosis of acute phase CD. A physical examination revealed abdominal tenderness and the presence of a palpable mass in the right ileac fossa. Laboratory test results revealed mild anemia (hematocrit 34.8%, hemoglobin 10.5 mg/dL, mean cell volume 73.3 fL, mean cell hemoglobin 24.2 pg) and low total albumin levels (6.1 g/dL). An abdominal contrast enhanced CT scan was performed, revealing a mass in the cecum and free peritoneal fluid (Figure 4). Colonoscopy was performed showing an obstructive mass in the ileo-cecal valve region, making further endoscopy impossible. Biopsies were taken and were inconclusive.

**Figure 5:**
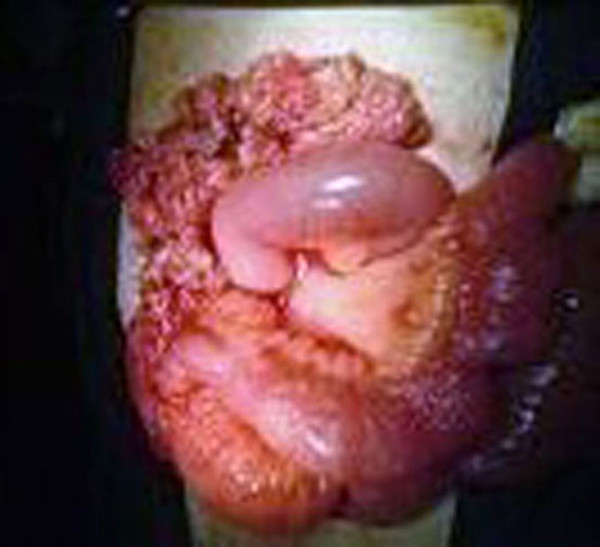
**Intra-operative picture showing tubercular adhesions of the omentum and mesenterium, and small intestine enlargement**.

On laparotomy, a large mass of the cecum and peritoneal implantations were revealed. Biopsies were taken and a bypass procedure (ileo-transverse colon anastomosis) was performed (Figure 5). Ziehl-Nielsen stain results were negative, but the culture and PCR results were positive for *Mycobacterium tuberculosis*. Anti-tuberculosis treatment was administered including rifampicin and isoniazid 300/150 mg twice a day, pyrizinamide 25 mg/kg/24 hours and vitamin B_6 _100 mg/day. At present (six months later) our patient remains free of symptoms.

## Discussion

The principle cause of PITB is *M. tuberculosis*. PITB may occur either as primary or secondary infection. The assumed routes of infection of the gastrointestinal tract are ingestion, hematogenous spread from the lungs, from infected lymph nodes and direct spread from adjacent organs. Rarely, *Mycobacterium bovis *is the cause due to unpasteurized milk and milk products [6]. Manifestations of gastrointestinal tuberculosis are variable. Symptoms are non-specific and include fever, night sweats, abdominal pain, weight loss and diarrhea. PITB is rarely a problem confronted by a surgeon. However, some of its complications can be a surgical issue. These complications are hemorrhage and obstruction, while fistulization and perforation also occur rarely [7,8].

More specifically, the ileo-cecal area is reported to be the area most commonly involved in intestinal tuberculosis [5,8-12]. The apparent affinity of the tubercule bacillus for lymphoid tissue and areas of physiological stasis, facilitating prolonged contact between the bacilli and the mucosa, may be the reasons for the ileum and cecum being the most common sites of disease. Other areas of the colon, besides the ileo-cecal area, represent the next more common site of tuberculous involvement of the gastrointestinal tract, usually manifesting as segmental colitis involving the ascending and transverse colon [5,12].

Colonic tuberculosis may present as an inflammatory stricture, hypertrophic lesions resembling polyps or tumors, segmental ulcers and colitis or, rarely, diffuse tuberculous colitis [6]. Diagnosis can be quite difficult since there are no specific clinical symptoms of large bowel tuberculosis and only a quarter of patients have chest radiographs showing evidence of active or healed pulmonary infection [5,8,12,13]. The colonoscopic features described in patients with colonic tuberculosis are transverse or linear ulcers, nodules, deformed ileo-cecal valve and cecum and presence of inflammatory polyps [5,12,14]. Furthermore, Misra *et al*. referred an additional finding of multiple fibrous bands arranged in a haphazard fashion, forming pockets [15].

With regard to the imaging findings in abdominal tuberculosis, the simple abdominal X-ray offers little or no help at all, as the findings of bowel obstruction or perforation that might be seen are non-specific, and the calcification of mesenteric lymph nodes, while rare, is unlikely to lead to the correct diagnosis if high awareness for the disease is not present. The main imaging techniques used are ultrasonography, CT, MRI and positron emission tomography. The common imaging features are: enlarged para-aortic nodes, asymmetric bowel wall thickening, ascites, inflammatory masses of the bowel wall lymph nodes and omentum, narrowing of the terminal ileum with thickening and gaping of the ileo-cecal valve, 'white bowel' sign due to lymphatic infiltration and 'sliced bread sign' due to fluid surrounding bowel caused by inflammation of the bowel wall [6].

The diagnostic procedure of choice for PTBC is colonoscopy and biopsy [15]. Apart from routine histology looking for caseating granulomas, appropriately stained slides should be prepared to look for acid-fast rods and biopsies should also be sent for culture [8]. Deep biopsies should be taken preferably from the margins of ulcerations, because tuberculus granulomas are often submucosal, as compared to the mucosal granulomas of Crohn's disease [8]; however, according to Misra *et al*. caseation may be absent or be present only in the lymph [15]. This finding is consistent with the fact that granulomas may not been seen in mucosal biopsies of nodules, ulcers or other lesions because they are mostly located in the submucosa of the tissue. Acid-fast bacilli have been reported in 50% to 100% of specimens from patients with intestinal tuberculosis, whereas in several reports acid-fast bacilli could not be detected on histological examination of the biopsy material [5,12,14]. Indeed, in our patients histology alone was unreliable since the results of the Ziehl-Nielsen stain for acid-fast bacilli were negative.

Culture of the biopsy material may be helpful [8], however, disappointing results with 0% detection of acid-fast bacilli have also been reported [5]. Culture sensitivity may be used, however, to determine the sensitivity of the bacilli to the drugs. This is becoming important because of the emergence of drug-resistant strains [15]. PCR analysis of biopsy specimens obtained endoscopically has been shown to be more sensitive than culture and acid-fast stains for the diagnosis of intestinal tuberculosis [13]. Sensitivity of this technique is 75% to 80% whereas specificity can reach 85% to 95%, depending on the type of specimen.

The differential diagnosis includes a broad spectrum of diseases. The clinical, radiological and endoscopic picture is most likely to be confused with neoplasms or CD, and infrequently with other conditions including amoeboma, Yersinia infection, gastrointestinal histoplasmosis and peri-appendiceal abscess [8]. Finally, the treatment of intestinal tuberculosis is mainly conservative, with surgery only required for complications.

## Conclusions

Tuberculosis is a re-emerging problem, concerning not only countries with high incidence, but Western countries as well. Constant demographic changes, the movement of populations, the incidence of HIV infection and the use of immunomodulator drugs mark the beginning of a new era with new challenges, where the clinical doctor is called upon to be highly aware and always up to date with new guidelines. Intestinal tuberculosis is a diagnostic puzzle, especially in low endemic countries where less experienced clinical doctors are only bibliographically familiar with the disease and its appearance, and clinical manifestation can imitate a broad spectrum of diseases. Attaining a cure can prove to be quite difficult as drug resistant strains seem to be met increasingly often. Surgery should be kept as the last resort and used only in complicated cases. It is our opinion that tuberculosis is not only a problem of underdeveloped countries, and that it is going to trouble the world further in the future.

## Consent

Written informed consent was obtained from both patients for publication of this case report and any accompanying images. A copy of the written consent is available for review by the Editor-in-Chief of this journal.

## Competing interests

The authors declare that they have no competing interests.

## Authors' contributions

AM: study design, drafting the manuscript and revising it critically. VNP: study design, drafting the manuscript. SP: study design, drafting the manuscript. TSP: study design, drafting the manuscript. AC: study design, drafting the manuscript. All authors read and approved the final manuscript.
